# CD62L (L-Selectin) Shedding for Assessment of Perioperative Immune Sensitivity in Patients Undergoing Cardiac Surgery with Cardiopulmonary Bypass

**DOI:** 10.1371/journal.pone.0053045

**Published:** 2013-01-03

**Authors:** Gabor Erdoes, Maria L. Balmer, Emma Slack, Istvan Kocsis, Lutz E. Lehmann, Balthasar Eberle, Frank Stüber, Malte Book

**Affiliations:** 1 University Department of Anesthesiology and Pain Therapy, Inselspital, Bern University Hospital, Bern, Switzerland; 2 University Bern, Department of Clinical Research, Gastroenterology, Maurice E Müller Laboratories, Bern, Switzerland; 3 Department of Microbiology, ETH Zürich, Zürich, Switzerland; 4 Department of Anesthesiology and Intensive Care, Semmelweis University of Medicine, Budapest, Hungary; Heart Center Munich, Germany

## Abstract

**Objective:**

To investigate the suitability of blood granulocyte and monocyte sensitivity, as measured by the quantity of different agonists required to induce CD62L shedding, for assessment of perioperative immune changes in patients undergoing cardiac surgery with cardiopulmonary bypass.

**Methods:**

Patients scheduled for aortocoronary bypass grafting or for valve surgery were included in this prospective observational study. Blood samples were drawn before anesthesia induction, directly after surgery and 48 hours after anesthesia induction. We determined the concentration of two different inflammatory stimuli – lipoteichoic acid (LTA) and tumor necrosis factor alpha (TNF) - required to induce shedding of 50% of surface CD62L from blood granulocytes and monocytes. In parallel monocyte surface human leukocyte antigen (HLA)-DR, and plasma interleukin (IL)-8, soluble (s)CD62L, soluble (s)Toll-like receptor (TLR)-2 and ADAM17 quantification were used to illustrate perioperative immunomodulation.

**Results:**

25 patients were enrolled. Blood granulocytes and monocytes showed decreased sensitivity to the TLR 2/6 agonist *Staphylococcus aureus* LTA immediately after surgery (p = 0.001 and p = 0.004 respectively). In contrast, granulocytes (p = 0.01), but not monocytes (p = 0.057) displayed a decreased postoperative sensitivity to TNF. We confirmed the presence of a systemic inflammatory response and a decreased immune sensitivity in the post-surgical period by measuring significant increases in the perioperative plasma concentration of IL-8 (p≤0.001) and sTLR (p = 0.004), and decreases in monocyte HLA-DR (p<0.001), plasma sCD62L (p≤0.001). In contrast, ADAM17 plasma levels did not show significant differences over the observation period (p = 0.401).

**Conclusions:**

Monitoring granulocyte and monocyte sensitivity using the “CD62L shedding assay” in the perioperative period in cardiac surgical patients treated with the use of cardiopulmonary bypass reveals common changes in sensitivity to TLR2/6 ligands and to TNF stimulus. Further long-term follow-up studies will address the predictive value of these observations for clinical purposes.

## Introduction

The clinical introduction of cardiopulmonary bypass (CPB) in 1953 by John Gibbon [1] opened a novel era in cardiac surgery, but confronted clinicians with previously unrecognized issues. Today, it is widely accepted that (i) exposure of blood to air and artificial surfaces in the extracorporeal circuit, (ii) ischemia-reperfusion injury and (iii) translocation of “pathogen associated molecular patterns” across the gut mucosal barrier are contributing factors to the activation of various molecular cascades resulting in systemic inflammatory response syndrome [2]. Clinical correlates range from mild myocardial depression or low systemic vascular resistance to life-threatening complications such as multi-organ failure. Besides this usually transient inflammatory response syndrome some patients acquire nosocomial infections (perioperative incidence 5% and 21% respectively [3], [4]) with influence on morbidity and mortality [4]–[6].

To understand the effects of CPB on the inflammatory system, a wide spectrum of laboratory and clinical studies have been performed. Effects of coronary artery bypass grafting performed off-pump (OPCABG) and on-pump (CABG) have been compared in a review [7]. This study identified a broad network of molecules involved in CPB-induced immunomodulation, for example, tumor necrosis factor alpha (TNF), interleukin (IL)-1, IL-6, IL-8, and IL-10 were found to be increased in CABG patients compared to OPCABG patients postoperatively [8]–[10]. Moreover, complement activation was higher when CABG had been performed [11]–[13]. However, taking into account that the involved markers reach peak plasma levels at different clinical stages, comparisons made on inflammatory markers alone might be misinterpreted [14], [15]. For this reason it is desirable to assess perioperative immune alterations not exclusively by measuring the concentration of individually selected key mediators at different time points, but also at a functional level, by assessing the sensitivity or responsiveness of inflammatory effector cells.

One method to quantify functional changes in immunity for their relevance in differentiating patient groups makes use of a concept previously employed to diagnose genetic defects in Toll-like receptor (TLR) signaling in a pediatric population by assessing cleavage of membrane-bound CD62L from granulocytes [16]. CD62L is an adhesion molecule present on the surface of blood granulocytes, inflammatory monocytes and naïve T cells. Upon activation of these cell types, CD62L is very rapidly enzymatically cleaved from the cell surface [17]–[19]. CD62L-cleavage can be quantified per cell within whole unfractionated human blood by loss of staining with an antibody specific to the shed portion of the molecule. By stimulating whole blood with a dose-titration of pro-inflammatory stimuli (TLR ligands, TNF) it is possible to calculate ligand concentrations required to induce shedding of 50% of surface CD62L from monocytes and granulocytes. This method provides a quantitative measurement of granulocyte and monocyte sensitivity that could be easily compared between patients.

The aim of this pilot study was to investigate whether sensitivity of granulocytes and monocytes to microbial and cytokine stimuli, as monitored by concentrations required to induce CD62L shedding, is modulated in cardiac surgical patients in the post-CPB period. Parallel quantification of the perioperative cytokine response (IL-8), human leukocyte antigen (HLA)-DR expression and the assessment of soluble plasma factors (sCD62L, sTLR-2) and ADAM17 activity has been performed to obtain further information on perioperative immunomodulation for comparison with “CD62L shedding” results.

## Materials and Methods

### Patients

The study was approved by the institutional ethical review board (approval number: 041/09) and patients were enrolled after written informed consent. All patients underwent elective cardiac surgery procedures with the use of CPB. The inclusion criteria were: age ≥18 years and elective coronary or valve surgery. The exclusion criteria were: active infection, active liver disease, renal failure or steroid use. The investigation was performed as a single center pilot prospective observational study in a university hospital. Patients were recruited consecutively in order of their date of surgery.

### Anesthesia and Surgery

All patients were anesthetized according to the clinical routine of the department. After premedication with lorazepam, general anesthesia was induced with midazolam, sufentanil and etomidate, muscular relaxation was achieved with pancuronium. Intraoperatively and on CPB anesthesia was maintained using a combination of isoflurane and continuous sufentanil infusion. After admission to postoperative intensive care a continuous infusion of propofol and boluses of fentanyl were used until patient`s extubation. All patients obtained 1.5 g cefuroxim preoperatively and after termination of CPB.

Conventional extracorporeal circulation (CECC) was used for all cases except isolated CABG surgery, which was managed using minimized extracorporeal circulation (MECC). CECC employed a standard circuit which consisted of tubings, a hollow-fiber membrane oxygenator, a roller pump, an arterial filter and a cardiotomy reservoir. The system was primed with a mixture of crystalloid (1000 ml) and colloid (800 ml) solution. The MECC consisted of a closed and preconnected system with a hollow-fiber membrane oxygenator and a centrifugal pump, which was primed with 600 ml of crystalloid solution. A neutrophil filter was not implemented in any of the bypass systems.

Cardiac surgery was performed using standard operation techniques. After median sternotomy a bolus of heparin was given (CECC: 500 IU/kg; MECC 400 IU/kg) prior to cannulation of the ascending aorta. The target activated clotting time (ACT_kaolin_) was >480 seconds (ACT Plus™, Medtronic Ltd., MN, USA). Furthermore, all patients received a bolus of tranexamic acid (30 mg/kg) followed by a continuous infusion (15 mg/kg/h) until the end of sternal closure. Once the CPB was initiated the aorta was cross-clamped and antegrade low-volume crystalloid cardioplegia (Cardioplexol, Laboratorium Dr. G. Bichsel AG, Interlaken, Switzerland) followed by high-potassium cold blood cardioplegia was delivered into the coronary arteries. After weaning from CPB heparin was reversed with protamin in the ratio 1∶1 with regard to the initial heparin bolus. Finally, all patients were transferred to the intensive care unit.

### Blood Sampling and Preparation

Blood sampling of 5 ml whole blood was performed at three time points in a heparinized and a citrate collection tube which were immediately transported to the laboratory. We chose citrate blood only for the CD62L-shedding assay as it lets us take into account the effects of procoagulatory signaling which is critical in innate immune activation. Furthermore, with the use of citrate, which is a weak Ca^2+^ chelator, we reduced the removal of calcium minimizing interactions with TLR signaling cascade.

The first sample (serving as control) was drawn after the radial arterial cannulation before induction of anesthesia. The second, at the beginning of the sternal wiring. The third, 48 hours after the first blood sampling.

### CD62L-shedding Assay

Twenty-five µl citrate whole blood was stimulated with 25 µl of six 10-fold dose-titrations starting at 10 µg/ml (LTA, Invivogen, San Diego, USA), 20 ng/ml (TNF, R&D). After 45 minutes incubation at 37°C/5% CO_2_, cells were washed in PBS/BSA 1% and stained with 25 µl PBS/BSA 1% containing APC-anti-human CD33 (*allowing identification of both granulocytes and monocytes in combination with side-scatter measurement, and remaining stable during stimulation* [*20*]) and FITC-anti-human CD62L antibodies (Biolegend, San Diego, USA) for 15 minutes. Red blood cells were lysed in 200 µl FACS Lysis solution (BD Biosciences, San Diego, USA) and acquired on a FACSArray SORP (BD Biosciences, San Diego, USA). Blood monocytes and granulocytes were distinguished on the basis of CD33 expression and side scatter using FlowJo Software (Tree Star Inc., Ashland, USA). Median FITC fluorescence intensity is computed for each sample’s granulocytes and monocytes and plotted against dilution factor to enable parallel analysis of multiple agonists. Four-parameter curves are fitted using non-linear regression and LogEC50 values were extracted, corresponding to the dilution factor giving 50% granulocyte or monocyte CD62L-shedding. The corresponding ligand concentration was calculated from the dilution factor and plotted for each ligand.

### IL-8 ELISA

IL-8 levels in plasma were quantified using the BD OptEIA^tm^ human IL-8 ELISA kit II (BD Bioscience, San Jose, CA, USA) according to the manufacturer’s instructions. All samples were diluted 1∶3 before loading onto the plate and measured in duplicates, as were all standards and blanks. The detection range (standard curve) was 3.1 to 200 pg/ml. Intra- and interassay variation ranged from 4 to 5.5 and 3.2 to 3.4%CV respectively (information provided by manufacturer).

### HLA-DR

Fifty microlitre heparinized whole blood were stained with 20 µl Quantibrite™ anti-human-HLA-DR PE(phycoerythrin)/anti-monocyte PerCP (peridinin chlorophyll protein)-Cy5.5 (anti-human-CD14 and anti-human-CD64) (BD Biosciences, San Diego, USA) at room temperature in the dark, for 25 minutes. Red blood cells were subsequently lysed with FACS lysing solution (BD Biosciences, San Diego, USA) for five to ten minutes and washed twice with phosphate buffered saline solution (Sigma-Aldrich, St. Louis, USA) and fixed with 400 µl 4% paraformaldehyde. One unstained sample was treated in the same manner. Fluorescence intensity of the samples was measured in duplicates on an LSR II Cytometer (BD Biosciences, San Diego, USA) using the software CellQuest (BD Biosciences, San Diego, USA). 500–1000 monocyte events were recorded. For quantification of the signal Quantibrite™ PE beads (BD Biosciences, San Diego, USA) were acquired with each measurement. FACS data was analyzed with FlowJo v. 7.5 (Tree Star Inc., Ashland, USA) by gating for CD14 and CD64 positive cells (monocytes). The HLA-DR PE channel was calibrated using the data from the PE Beads, which allows the correlation of fluorescence intensity to the mean number of PE molecules per cell. The results were recorded as the geometric mean of the calibrated PE channel fluorescence intensity of each sample.

### sCD62L (sL-selectin) ELISA

Plasma samples were diluted 1∶200 and the sL-selectin concentration was measured with a human sL-selectin platinum ELISA kit (eBioscience, Vienna, Austria) according to the manufacturer`s instructions. All standards, samples and controls were measured in duplicates. The detection range (standard curve) was 0.4 to 25 ng/ml. Intra- and interassay variation was 3.7 and 4.2%CV respectively, as declared by the manufacturer.

### sTLR-2

sTLR-2 plasma concentrations were detected using a human TLR-2 ELISA kit (Cusabio, Wuhan, China). Samples were diluted 1∶3 and measured together with standards and blanks in duplicate. The detection range (standard curve) was 0.312–20 ng/ml, intra- and interassay variation was given as 8 and 10%CV by the manufacturer.

### ADAM 17 (TACE) ELISA

All samples were measured undiluted and in duplicates using a TACE human ELISA kit (Abcam, Cambridge, UK) according to the manufacturer’s instructions. The detection range (standard curve) was 78.15–5000 pg/ml, intra- and interassay variation was given as 10 and 12%CV by the manufacturer.

Absorption was read at 450 nm for all measurements with an Elx800 Microplate reader (Biotek Instruments) and evaluated with Gen5 Software (v1.09). The blank absorption value was subtracted from all standards and samples. These values were then used to draw a standard curve from which the concentrations of the samples were derived. A coefficient of variation below 20% was considered acceptable.

### Statistical Analysis

Categorical data are given as integral number, percentage or mean and standard deviation. For comparisons between groups or repeated measures, data normality was tested by the Shapiro-Wilk normality test. Non-parametric testing was performed by Friedman Repeated Measures ANOVA on ranks with the Tukey post-hoc test for all pairwise multiple comparisons procedures. These data are presented as medians with interquartile ranges. The Pearson product moment correlation has been used for analyses between the tests quantifying immune sensitivity. For all statistical results a p-value of <0.05 was considered as significant. All statistical analyses were performed using the SigmaPlot software version 12.0 (Systat Software Inc., San Jose, CA, USA).

## Results

### Demographics of the Study Population and Procedure Characteristics

Twenty-five consecutive patients were included in the study. Fourteen patients underwent CABG surgery, 9 patients underwent valve surgery (VS) and two patients received combined surgery (CABG and VS). Eleven patients were treated using CECC whereas fourteen patients underwent perfusion using MECC (TABLE 1.).

**Table 1:** Basic clinical and procedural characteristics according to the type of surgery or the type of ECC.

Type of surgery	All	CABG	VS	CABG+VS	p*
Patients	25	14	9	2	
Age, years	67±12	71±10	63±12	54±19	ns
Female, sex	12 (48)	6 (43)	5 (55)	1 (50)	ns
Diabetes	9 (36)	7 (50)	2 (22)	0 (0)	ns
EuroScore (additive)	6±3	5±2	6±4	6±7	ns
Duration ofsurgery, min	230±58	228±41	232±77	235±106	ns
ECC time, min	83±42	60±14	112±51	105±49	0.003
LOS, days	9.7±2.7	9.4±2	10.3±3.8	9±0	ns
In-hospital mortality	0 (0)	0 (0)	0 (0)	0 (0)	ns
**Type of ECC**		**CECC**	**MECC**		**p** *
Patients		11	14		
Age, years		61±12	71±10		ns
Female, sex		6 (54)	6 (43)		ns
Diabetes		2 (18)	7 (50)		ns
EuroScore (additive)		7±4	5±2		ns
Duration ofsurgery, min		233±77	228±41		ns
ECC time, min		111±48	60±14		<0.001
LOS, days		10±3	9±2		ns
In-hospital mortality		0 (0)	0 (0)		ns

Values are presented as total number of patients (percent) or mean ± standard deviation.

* p- values >0.05 considered as non significant (ns).

Definitions: CABG, coronary artery bypass grafting; VS, valve surgery; ECC, extracorporeal circulation; CECC, conventional extracorporeal circulation; MECC, minimized extracorporeal circulation; LOS, length of postoperative hospital stay.

### CD62L Shedding of Granulocytes and Monocytes after Stimulation with LTA

**Figure 1:**
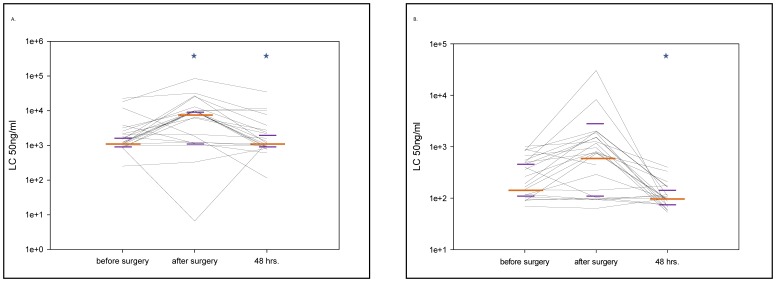
Granulocyte and monocyte sensitivity by lipoteichoic acid stimulation. 1A and B. Granulocyte (A) and monocyte (B) sensitivity by lipoteichoic acid (LTA) stimulation was modified in the perioperative period. The figure shows the median values (long orange line) with interquartile ranges (short purple lines) at the different sampling points (*p<0.05 compared with the previous sampling point). Each black line represents data from an individual patient. The CD62L shedding assay was quantified by measuring the ligand concentration (LC)50. In the study setting LC50 represented the concentration of the stimulant which caused the shedding of 50% of the CD62L molecules from the surface of the cell type of interest. Thus, in the presented figure, higher values of LC50 represent a loss of sensitivity to stimulation with this particular agonist.

A ten-fold increase in the concentration of LTA from the gram positive bacterium *Staphylococcus aureus* required to activate both granulocytes (p = 0.001) and monocytes (p = 0.004) was apparent immediately post-surgery in a large fraction of patients (FIGURE 1A and B). In the majority of patients, sensitivity is recovered 48 hours post-surgery (TABLE 2.).

**Table 2:** Granulocyte and monocyte activation with different inflammatory stimuli.

	before surgery	after surgery	48 hrs.	p*
LTA				
granulocytes	1225 [999;2364]	9602 [1242;10000]	1177 [916;2748]	0.001
monocytes	191 [112;508]	780 [113;1535]	97 [74;164]	0.004
TNF				
granulocytes	2 [2]; [9]	20.0 [2;28.5]	3 [2]; [5]	0.01
monocytes	1 [0;2]	1 [1]; [3]	2 [1]; [3]	0.057
HLA				
CD14/CD64	19204 [12740;24268]	9694 7907;11039]	6969 [4303;11976]	0.001

Values are presented as median and interquartile ranges [25^th^;75^th^].

* p- values >0.05 considered as non significant.

Definitions: LTA, lipoteichoic acid; TNF, tumor necrosis factor; HLA, human lymphocyte antigen; CD14/64, cluster of differentiation 14/64 monocytes.

### CD62L Shedding of Granulocytes and Monocytes after Stimulation with TNF

**Figure 2:**
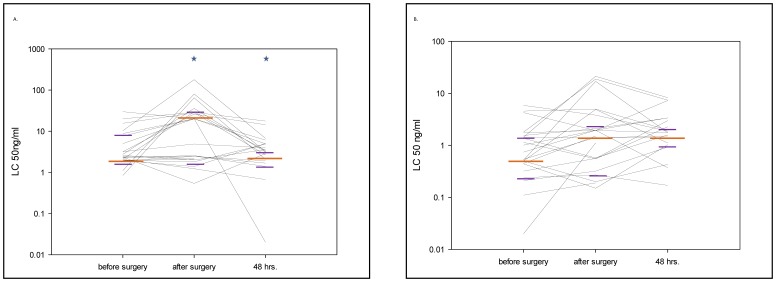
Granulocyte and monocyte sensitivity by tumor necrosis factor alpha stimulation. 2A and B. Granulocyte (A) and monocyte (B) sensitivity by tumor necrosis factor (TNF) stimulation. The figure shows the median values (long orange line) with interquartile ranges (short purple lines) at the different sampling points (*p<0.05 compared with the previous sampling point). Each black line represents data from an individual patient. The CD62L shedding assay was quantified by measuring the ligand concentration (LC)50. In the study setting LC50 represented the concentration of the stimulant which caused the shedding of 50% of the CD62L molecules from the surface of the cell type of interest. Thus, in the presented figure, higher values of LC50 represent a loss of sensitivity to stimulation with this particular agonist.

We observed a roughly 10-fold decreased sensitivity of granulocytes to TNF stimulation immediately post-surgery, as revealed by a 10-fold increased concentration of TNF required to produce 50% loss of CD62L-staining on the cell surface (p = 0.01, TABLE 2.). In contrast to changes in LTA sensitivity, the change in TNF sensitivity was not clearly observed in monocytes stimulated in the same blood samples (p = 0.057, FIGURE 2A and B). The change in TNF sensitivity was clearly heterogeneous between patients, i.e. approximately 50% of patients showed this change in sensitivity, whereas others maintained sensitivity throughout the operation period.

### Flow Cytometric Measurement of Monocytic HLA-DR Expression

**Figure 3:**
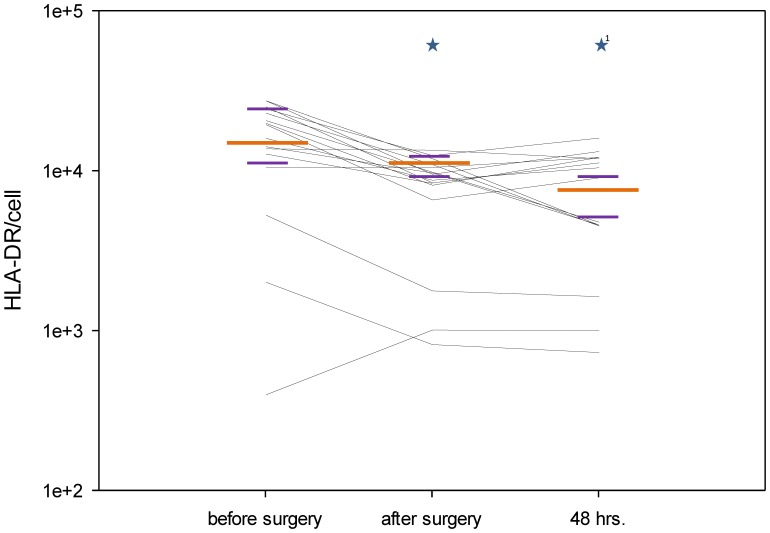
Monocytic HLA-DR expression. HLA-DR molecules per CD14 and CD64 positive cell calculated perioperatively. The figure shows the median values (long orange line) with interquartile ranges (short purple lines) at the different sampling points (*p<0.05 compared with the previous sampling point; ^1^*p<0.05 compared with the first sampling point). Each black line represents data from an individual patient.

In seventeen of the twenty-five patients analyzed for granulocyte and monocyte sensitivity, HLA-DR surface expression was quantified in CD14 and CD64 double-positive monocytic cells. This analysis showed a perioperative decrease in median density of surface HLA-DR on monocytes in patients treated with CPB (p = 0.001, TABLE 2., FIGURE 3).

### Association of HLA-DR Expression Data with CD62L Shedding to LTA and TNF

Regression analyses within our sample detected no significant statistical associations between the three sensitivity measures shown to change in the perioperative period (LTA/TNF: r = 0.10, p = 0.40; LTA/HLA-DR: r = −0.21, p = 0.078; TNF/HLA-DR: r = −0.13, p = 0.28).

### Perioperative Plasma Levels of IL-8, sCD62L, sTLR-2 and ADAM17

These analyses were performed in a subset of patients (18/25) due to limited sample volume.

**Figure 4:**
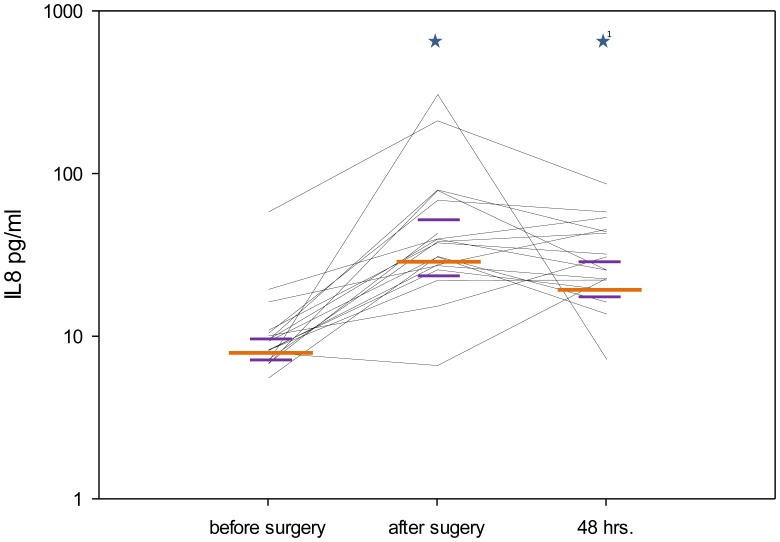
Perioperative plasma levels of IL-8. IL-8 concentration (pg/ml) in the perioperative course. The figure shows the median values (long orange line) with interquartile ranges (short purple lines) at the different sampling points (*p<0.05 compared with the previous sampling point; ^1^*p<0.05 compared with the first sampling point). Each black line represents data from an individual patient.

**Figure 5:**
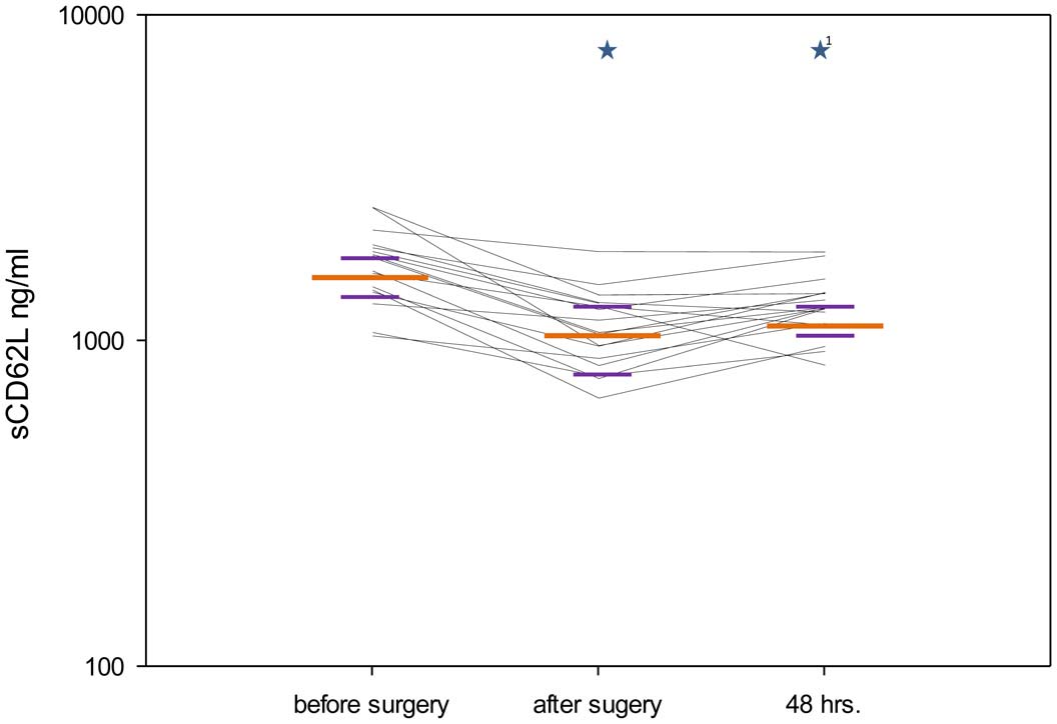
Perioperative plasma levels of sCD62L. sCD62L (ng/ml) concentration in the perioperative course. The figure shows the median values (long orange line) with interquartile ranges (short purple lines) at the different sampling points (*p<0.05 compared with the previous sampling point; ^1^*p<0.05 compared with the first sampling point). Each black line represents data from an individual patient.

IL-8 plasma levels significantly increased after surgery (p≤0.001) and after 48 hours (p≤0.001) when compared with the preoperative values (FIGURE 4), whereas sCD62L plasma levels significantly decreased after surgery (p≤0.001) and after 48 hours (p≤0.001) in comparison with the preoperative levels (FIGURE 5). sTLR-2 showed a significant decrease between the sampling points “after surgery” and “48 hours.” (p = 0.004, TABLE 3). The analysis of ADAM17 activity did not show significant differences between the different time points (p = 0.401), but a trend of the medians was apparent towards higher values postoperatively and after 48 hours (TABLE 3).

**Table 3:** sTLR-2 and ADAM17 course during the observational period.

	before surgery	after surgery	48 hrs.	p*
sTLR-2	1.4 (1.2;2.4)	2.5 (1.9;3.6)	0.8 (0.6;1.2)	0.004
ADAM17	436 (227;849)	574 (274;1769)	761 (402;1145)	0.401

Values are presented as median and interquartile ranges (25^th^;75^ th^).

* p- values >0.05 considered as non significant.

Definitions: sTLR-2, soluble form of Toll-like receptor-2; ADAM17, ADAM metalloproteinase domain 17 (also called TACE – tumor necrosis factor-α-converting enzyme).

### CD62L Shedding between Different Cardiopulmonary bypass Methods and Surgical Procedures

Changes in sensitivity of granulocytes and monocytes to LTA stimulation and granulocytes to TNF stimulation were observed similarly in patients undergoing both CECC and MECC. Additionally, no difference was seen between patients undergoing CABG or VS (TABLE 4).

**Table 4:** Granulocyte and monocyte stimulation in different bypass techniques and surgeries**.**

		CECC	MECC	p	CABG	VS	p
**LTA**							
before surgery	granulocytesmonocytes	1770 [966;2564]264 [119;494]	1112 [946;2342]174 [90;575]	nsns	1112 [859;2133]319 [93;634]	1770 [1087;7319]191 [117;379]	nsns
aftersurgery	granulocytesmonocytes	10000 [1242;12951]804 [103;2000]	6209 [1211;10000]769 [185;1429]	nsns	10000 [1526;10000]870 [355;1918]	9205 [1195;22629]290 [98;1397]	nsns
48 hrs.	granulocytesmonocytes	1291 [986;2748]111 [74;175]	1086 [830;3758]96 [59;109]	nsns	1086 [850;2973]96 [63;110]	1618 [1013;5220]116 [74;192]	nsns
**TNF**							
before surgery	granulocytesmonocytes	2 [2]; [8]1 [0.5;2]	3 [2]; [12]1 [0;2]	nsns	3 [2]; [10]1 [0;1.5]	2 [2]; [9]1[0;3]	nsns
aftersurgery	granulocytesmonocytes	20 [2]; [36]2 [1]; [4]	12 [2]; [25]1 [0;4]	nsns	4 [2]; [22]1 [0;3]	28 [2;50]2 [1.5;4]	nsns
48 hrs.	granulocytesmonocytes	3 [2]; [5]2 [1]; [2]	3.6 [1]; [6]2 [1]; [7]	nsns	3 [1]; [5]2 [1]; [6]	3 [2]; [4]2[1]; [2]	nsns

Values are presented as median and interquartile ranges [25^th^;75^th^]. ns =  non significant difference.

Definitions: CECC, conventional extracorporeal circulation; MECC, minimized extracorporeal circulation; CABG, coronary artery bypass grafting; VS, valve surgery; LTA, lipoteichoic acid; TNF, tumor necrosis factor alpha.

## Discussion

This pilot study investigated perioperative immunomodulation in cardiac surgery patients treated with CPB, using a novel CD62L shedding assay. In contrast to methods quantifying cytokine levels in serum or cell surface phenotypes, the CD62L-shedding assay allowed direct quantification of the immunologically relevant function of blood granulocytes and monocytes. Moreover, the comparison of our observations to established methods of quantifying immune change indicate that monitoring of perioperative immunomodulation by the CD62L shedding assay could contribute to a more complete interpretation of perioperative immune response by assessing the sensitivity of inflammatory effector cells to microbial stimulation on a functional level.

The shedding of the extracellular domain of the CD62L receptor is detectable minutes after cell activation [17]–[19]. Previous studies have measured CD62L shedding by ELISA, but such methods either cannot identify the cellular source of shed CD62L if carried out on whole blood, or require lengthy cell purification [21]. Instead, we have made use of a technique developed by *Slack et al.* that uses the cleavage of the membrane bound CD62L molecules as a surrogate parameter for early cell activation (manuscript in preparation). In contrast to previous studies examining the diagnostic value of changes in CD62L expression levels during various disease states [11], [21]–[23], our data is entirely independent of absolute CD62L-expression levels [24], [25]. In this regard the CD62L shedding assay is a novel method to monitor neutrophil responsiveness using a range of agonist concentrations from sub-saturating to fully saturating and fitting 4-parameter curves to calculate a sensitivity independently of the absolute receptor load on the cell surface. This avoids potential loss of information when “close-to-saturating” doses are applied, potentially revealing no altered absolute CD62L level despite a change in sensitivity. Therefore, our method provides different information regarding cell functionality from the information gleaned from the absolute receptor number per cell directly *ex vivo*.

As showed by Pillay et al. the identification of human neutrophil subsets can further contribute to a better understanding of mechanisms underlying immune changes following endotoxin challenge or severe injury [26]. As our LC50 calculations provide an average sensitivity to stimulation over all CD33-intermediate, side-scatter high cells, any augmentation of a neutrophil subset falling within this definition would almost certainly contribute to an altered LC50 (since we averaged over all neutrophils) and may be part of the mechanism underlying altered sensitivity. A further contributor to the mechanism of altered myeloid cell sensitivity could also be *in vivo* activation of myeloid cells by the surgical procedure, possibly including some *in vivo* shedding of CD62L. As the LC50 calculation relies upon the difference in CD62L surface staining between unstimulated cells and those cells stimulated with a saturating dose of agonist, a lower “unstimulated” surface CD62L level may indeed affect the LC50, but most likely less than signaling changes induced by the pre-activation itself. Interestingly, elevated soluble TLR2 levels in plasma correspond to decreased sensitivity to the TLR2/6 agonist LTA, suggesting that an important modulator of sensitivity to TLR2 agonists post-surgery may be secretion of the soluble receptor form. However, due to limited marker usage, a full investigation into underlying mechanisms would require a follow-up study to elucidate.

Our presented method thus evaluated the sensitivity of monocytes and granulocytes to an *ex vivo* microbial stimulation by quantifying the LC50 value, always defined as the concentration of agonist applied ex vivo that elicits a 50% drop in CD62L MFI. A variety of underlying mechanisms, including presence of soluble antagonists, cell activation and appearance of novel subsets, may alter the observed LC50, making this potentially a useful read-out of a broad range of immune changes.

Given the preliminary nature of this pilot study and the size and diversity of the cohort studied, it is surprising that such marked differences in granulocyte and monocyte sensitivity could be observed immediately post-surgery. However, some clues to the relevance may come from examining why changes in responsiveness are observed specifically to LTA and TNF. Explanations could either involve modulation of receptor and signaling component expression in pre-existing cells by the blood milieu during surgery, or release of less mature granulocytes and monocytes into the circulation from bone marrow stores which may have a slightly different expression profile of receptors and signaling components for the TLR2/6 and TNF receptor pathways.

A well-established phenomenon in immunobiology is that of endotoxin tolerance, i.e. exposure to Lipopolysaccharide from gram negative *Escherichia coli* is followed by suppressed responsiveness to stimulation with the same agonist [27]. Diacylated lipopeptides that form the ligands for the TLR2/6 receptor complex are common major components of the cell walls of gram positive bacteria. Hence, it may be relevant to note that Hamers and colleagues found live culturable bacteria in around 1% of the priming circuit cultures and 5.6% of the CBP blood cultures [28]. About 80% of the positive priming circuit cultures and about 70% of the CBP blood cultures were infected with gram positive cocci. Such bacterial species are very common skin commensal bacteria that the patient may be exposed to during these long surgical procedures. However, for this to explain our data, the recovery rate of live bacteria would have to massively underestimate the actual exposure as almost all of the 25 patients examined showed loss of sensitivity to TLR2/6 ligands compare to a predicted 1–6% contamination rate of patient blood. An alternative explanation is suggested by the observed post-surgical myelocytosis in patient blood. The bone marrow harbors a massive store of blood granulocytes and monocytes, with only a small percentage of these cells present in the circulation [29]. Activation of damage-sensing pathways by the surgical procedure and blood contact to foreign surfaces is a strong stimulus for bone marrow myeloid pool mobilization, releasing a large number of “fresh” granulocytes and monocytes into the peripheral circulation. It is highly possible that newly released cells require several hours to develop the full repertoire of signaling receptors and transducers present on fully mature granulocytes and monocytes, or that the results of a final maturation step in the “activated” surgical milieu produces a different mature state to that developed under resting conditions. Thus, altered sensitivity to LTA and TNF is likely to be the result of activator signals received by the patient immune system, either due to exposure to contaminating gram positive bacteria, or directly due to the surgical procedure. Likewise, the severity of deviation in sensitivity may well be relevant during the postoperative period when infections with gram-positive bacteria such as *Staphylococcus aureus* are a major concern. Unfortunately, our small study allows no pertinent conclusions due to small sample size, diversity of surgical procedures and perfusion techniques. Moreover, our pilot feasibility study was not designed in this direction.

To validate the presented CD62L shedding-assay and to gain a more complete insight into the perioperative inflammatory state, in particular the change in neutrophil response, the application of established methods and the analysis of multiple markers would be desirable as proposed by Pillay [24]. For this reason the perioperative course of IL-8, HLA-DR, sCD62L, sTLR-2 and ADAM17 has been investigated additionally to CD62L shedding assay.

The measured IL-8 results are in accordance with other groups [30]–[32] and confirm a proinflammatory response post-CPB in our cohort. However, the IL-8 course is more of general character being affected by a great variability of intraoperative factors and does not allow conclusions about the functional immune status in the patients. Likewise, our HLA-DR results confirmed previously observed decrease in expression levels post-CPB. Moreover the results agree with published absolute molecule numbers per cell preoperatively, in the post-surgical period and after 48 hours [33]. The lack of any mutual association of HLA-DR expression data and sensitivity to LTA or TNF in our cohort may well indicate that these three responses represent independent aspects of post-surgical immune function. At this stage, however, we cannot exclude that this is simply due to under-powering of our study.

The perioperative course of sCD62L plasma concentration as a further surrogate marker of in vivo neutrophil activation was in line with other investigations [34]–[35] The reduced levels of sCD62L were presumed to reflect the reduced CD62L shedding in the postoperative period and after 48 hours, which is highly suggestive of a (temporary) decrease of circulating neutrophil activity [36] in keeping with our observations for decreased neutrophil sensitivity to TNF and lipoteichoic acid.

The sTLR-2 increase after the surgical procedure was accompanied by an increase of the LC50 values for LTA, as determined with the “CD62L-shedding assay” reflecting decreased granulocyte and monocyte reactivity. Also, at the time point “48 hrs.” the decreased sTLR-2 values were accompanied by decreased LC50 values showing normalization of immune reactivity. Thus it is likely that the previously assumed role of sTLR-2 as a negative regulator of TLR-2 mediated signaling pathways [37] is a major contributor to the altered responsiveness to LTA observed post-surgery.

The analysis of ADAM17 confirms perioperative inflammation as measured by different markers in this study. However, we were not able to detect a direct correlation between ADAM17 and CD62L shedding pattern (ADAM17 levels remained still high after 48 hrs.) This result maybe reflects previous findings on ADAM17 regulating CD64L`s surface density (which we did not directly quantify) but not the cleavage (which was the major read-out used to determine LC50 measurements of sensitivity) [38].

In conclusion, monitoring granulocyte and monocyte sensitivity using the “CD62L shedding assay” in the perioperative period in cardiac surgical patients undergoing cardiopulmonary bypass reveals common changes in sensitivity to TLR2/6 ligands and to TNF stimulus. Our data support the further investigation of this novel assay to provide complementary information to currently established methods for the interpretation of perioperative immune responsiveness.

Further long-term follow-up studies with larger and more homogenous cohorts will help to address the predictive value of these observations for clinical purposes and to elucidate whether the described changes relate to the exact surgical procedure or inter-individual variation.

### Limitations of the Study

This study has several limitations. The main limitation is probably its small sample size with a heterogeneous patient population undergoing different cardiac surgical operations using different cardiopulmonary bypass techniques. As a consequence, the study cannot draw pertinent conclusions on the diagnostic relevance of our observations. However, the current study was intended to investigate the feasibility of CD62L-shedding measurements in assessment of patients during surgical procedures. Follow-up studies will now be able to take this observation of feasibility to evaluate the effect of cardiac surgery on the immune responsiveness and to discriminate differences in the peri-operative immune sensitivity between variable perioperative conditions.

## References

[1] Gibbon JH Jr (1954) Application of a mechanical heart and lung apparatus to cardiac surgery. Minn Med 37: 171–185. 13154149

[2] Morisset R, Agbaba O, Beaudet R, Adrien A (1977) Effect of open heart surgery on the defense mechanisms against bacterial infections. Can Med Assoc J 116: 279–281. 319885 PMC1878929

[3] Michalopoulos A, Geroulanos S, Rosmarakis ES, Falagas ME (2006) Frequency, characteristics, and predictors of microbiologically documented nosocomial infections after cardiac surgery. Eur J Cardiothorac Surg 29: 456–460. 16481186 10.1016/j.ejcts.2005.12.035

[4] Kollef MH, Sharpless L, Vlasnik J, Pasque C, Murphy D, et el (1997) The impact of nosocomial infections on patient outcomes following cardiac surgery. Chest 112: 666–675. 9315799 10.1378/chest.112.3.666

[5] Falagas ME, Rosmarakis ES, Rellos K, Michalopoulos A, Samonis G, et al (2006) Microbiologically documented nosocomial infections after coronary artery bypass surgery without cardiopulmonary bypass. J Thorac Cardiovasc Surg 132: 481–490. 16935099 10.1016/j.jtcvs.2006.05.019

[6] Ryan T, Mc Carthy JF, Rady MY, Serkey J, Gordon S, et al (1997) Early bloodstream infection after cardiopulmonary bypass: frequency rate, risk factors, and implications. Crit Care Med 25: 2009–2014. 9403751 10.1097/00003246-199712000-00018

[7] Raja SG, Dreyfus GD (2005) Modulation of systemic inflammatory response after cardiac surgery. Asian Cardiovasc Thorac Ann 13: 382–395. 16304234 10.1177/021849230501300422

[8] Mazzone A, Gianetti J, Picano E, Bevilacqua S, Zucchelli G, et al (2003) Correlation between inflammatory response and markers of neuronal damage in coronary revascularization with and without cardiopulmonary bypass. Perfusion 18: 3–8. 12705643 10.1191/0267659103pf622oa

[9] Hazama S, Eishi K, Yamachika S, Noguchi M, Ariyoshi T, et al (2004) Inflammatory response after coronary revascularization: off-pump versus on-pump (heparin-coated circuits and poly2methoxyethyl-acrylate-coated circuits). Ann Thorac Cardiovasc Surg 10: 90–96. 15209550

[10] Wan IY, Arifi AA, Wan S, Yip JH, Sihoe AD, et al (2004) Beating heart revascularization with or without cardiopulmonary bypass: evaluation of inflammatory response in a prospective randomized study. J Thorac Cardiovasc Surg 127: 1624–1631. 15173716 10.1016/j.jtcvs.2003.10.043

[11] Wehlin L, Vedin J, Vaage J, Lundahl J (2004) Activation of complement and leukocyte receptors during on- and off pump coronary artery bypass surgery. Eur J Cardiothorac Surg 25: 35–42. 14690730 10.1016/s1010-7940(03)00652-3

[12] Diegeler A, Doll N, Rauch T, Haberer D, Walther T, et al (2000) Humoral immune response during coronary artery bypass grafting: A comparison of limited approach, “off-pump” technique, and conventional cardiopulmonary bypass. Circulation 102: III95–100. 11082370 10.1161/01.cir.102.suppl_3.iii-95

[13] Gu YJ, Mariani MA, van OW, Grandjean JG, Boonstra PW (1998) Reduction of the inflammatory response in patients undergoing minimally invasive coronary artery bypass grafting. Ann Thorac Surg 65: 420–424. 9485239 10.1016/s0003-4975(97)01127-2

[14] Pekna M, Hagman L, Halden E, Nilsson UR, Nilsson B, et al (1994) Complement activation during cardiopulmonary bypass: effects of immobilized heparin. Ann Thorac Surg 58: 421–424. 8067842 10.1016/0003-4975(94)92219-5

[15] Asimakopoulos G, Taylor KM (1998) Effects of cardiopulmonary bypass on leukocyte and endothelial adhesion molecules. Ann Thorac Surg 66: 2135–2144. 9930520 10.1016/s0003-4975(98)00727-9

[16] von Bernuth H, Ku CL, Rodriguez-Gallego C, Zhang S, Garty BZ, et al (2006) A fast procedure for the detection of defects in Toll-like receptor signaling. Pediatrics 118: 2498–2503. 17142536 10.1542/peds.2006-1845

[17] Griffin JD, Spertini O, Ernst TJ, Belvin MP, Levine HB, et al (1990) Granulocyte-macrophage colony-stimulating factor and other cytokines regulate surface expression of the leukocyte adhesion molecule-1 on human neutrophils, monocytes, and their precursors. J Immunol 145: 576–584. 1694883

[18] Spertini O, Freedman AS, Belvin MP, Penta AC, Griffin JD, et al (1991) Regulation of leukocyte adhesion molecule-1 (TQ1, Leu-8) expression and shedding by normal and malignant cells. Leukemia 5: 300–308. 1709244

[19] Zhao LC, Edgar JB, Dailey MO (2001) Characterization of the rapid proteolytic shedding of murine L-selectin. Dev Immunol 8: 267–277. 11785676 10.1155/2001/91831 PMC2276085

[20] Schütt C, Ringel B, Nausch M, Bazil V, Horejsi V, et al (1988) Human monocyte activation induced by an anti-CD14 monoclonal antibody. Immunol Lett 19: 321–327. 2468604 10.1016/0165-2478(88)90162-9

[21] Pavelkova M, Kubala L, Ciz M, Pavlik P, Wagner R, et al (2006) Blood phagocyte activation during open heart surgery with cardiopulmonary bypass. Physiol Res 55: 165–173. 15910174 10.33549/physiolres.930662

[22] Wehlin L, Vedin J, Vaage J, Lundahl J (2005) Peripheral blood monocyte activation during coronary artery bypass grafting with or without cardiopulmonary bypass. Scand Cardiovasc J 39: 78–86. 16097419 10.1080/14017430410004623

[23] Ilton MK, Langton PE, Taylor ML, Misso NL, Newman M, et al (1999) Differential expression of neutrophil adhesion molecules during coronary artery surgery with cardiopulmonary bypass. J Thorac Cardiovasc Surg 118: 930–937. 10534700 10.1016/s0022-5223(99)70064-4

[24] Pillay J, Hietbrink F, Koenderman L, Leenen LPH (2007) The systemic inflammatory response induced by trauma is reflected by multiple phenotypes of blood neutrophils. Injury 38: 1365–1372. 18061190 10.1016/j.injury.2007.09.016

[25] Videm V, Strand E (2004) Changes in neutrophil surface-receptor expression after stimulation with FMLP, endotoxin, interleukin-8 and activated complement compared to degranulation. Scand J Immunol 59: 25–33. 14723618 10.1111/j.0300-9475.2004.01351.x

[26] Pillay J, Kamp V, van Hoffen E, Visser T, Tak T, et al (2012) A subset of neutrophils in human systemic inflammation inhibits T cell responses through Mac-1. J Clin Invest 122: 327–36. 22156198 10.1172/JCI57990 PMC3248287

[27] Biswas SK, Lopez-Collazo E (2009) Endotoxin tolerance: new mechanismus, molecules and clinical significance. Trends Immunol 30: 475–487. 19781994 10.1016/j.it.2009.07.009

[28] Hamers LA, Linssen CF, Lance MD, Maessen JG, Weerwind P, et al (2011) Positive cultures from cardiopulmonary bypass: prevalence and relevance regarding postoperative infection. Eur J Cardiothorac Surg 40: 372–378. 21247775 10.1016/j.ejcts.2010.11.063

[29] Day RB, Link DC (2012) Regulation of neutrophil traffickingfrom the bone marrow. Cell Mol Life Sci 69: 1415–1423. 22045556 10.1007/s00018-011-0870-8 PMC11114822

[30] Ascione R, Lloyd CT, Underwood MJ, Lotto AA, Pitsis AA, et al. (2000) Inflammatory response after coronary revascularization with or without cardiopulmonary bypass. Ann Thorac Surg 69: 1198–1204 or. 10.1016/s0003-4975(00)01152-8 10800819

[31] Kawamura T, Wakusawa R, Okada K, Inada S (1993) Elevation of cytokines during open heart surgery with cardiopulmonary bypass: participation of interleukin 8 and 6 in reperfusion injury. Can J Anaesth 40: 1016–21. 8269560 10.1007/BF03009470

[32] Hambsch J, Osmancik P, Bocsi J, Schneider P, Tarnok A (2002) Neutrophil adhesion molecule expression and serum concentration of soluble adhesion molecules during and after pediatric cardiovascular surgery with or without cardiopulmonary bypass. Anesthesiology 96: 1078–85. 11981146 10.1097/00000542-200205000-00009

[33] Docke WD, Hoflich C, Davis KA, Rottgers K, Meisel C, et al (2005) Monitoring temporary immunodepression by flow cytometric measurement of monocytic HLA-DR expression: a multicenter standardized study. Clin Chem 51: 2341–2347. 16214828 10.1373/clinchem.2005.052639

[34] Williams HJ, Rebuck N, Elliott MJ, Finn A (1977) Changes in leukocyte counts and soluble intercellular adhesion molecule-1 and E-selectin during cardiopulmonary bypass in children. Perfusion 13: 322–7. 10.1177/026765919801300507 9778716

[35] Blume ED, Nelson DP, Gauvreau K, Walsch AZ, Plumb C, et al (1997) Soluble adhesion molecules in infants and children undergoing cardiopulmonary bypass. Circulation 96 suppl: II352–7. 9386123

[36] Spertini O, Schleiffenbaum B, White-Owen C, Ruiz P Jr, Tedder TF (1992) ELISA for quantitation of L-selectin shed from leukocytes in vivo. J Immunol Methods 156: 115–123. 1385536 10.1016/0022-1759(92)90017-n

[37] Raby AC, Le Bouder E, Colmont C, Davies J, Richards P, et al (2009) Soluble TLR2 reduces inflammation without compromising bacterial clearance by disrupting TLR2 triggering. J Immunol 183: 506–517. 19542461 10.4049/jimmunol.0802909

[38] Li Y, Brazzell J, Herrera A, Walcheck B (2006) ADAM17 deficiency by mature neutrophils has differential effects on L-selectin shedding. Blood 108: 2275–2279. 16735599 10.1182/blood-2006-02-005827 PMC1895557

